# Radiopharmaceutical Validation for Clinical Use

**DOI:** 10.3389/fonc.2021.630827

**Published:** 2021-03-03

**Authors:** Charles A. Kunos, Rodney Howells, Aman Chauhan, Zin W. Myint, Mark E. Bernard, Riham El Khouli, Jacek Capala

**Affiliations:** ^1^ Cancer Therapy Evaluation Program, National Cancer Institute, Bethesda, MD, United States; ^2^ Division of Medical Oncology, Department of Internal Medicine, University of Kentucky, Lexington, KY, United States; ^3^ Department of Radiation Medicine, University of Kentucky, Lexington, KY, United States; ^4^ Division of Nuclear Medicine and Molecular Imaging, Department of Radiology, University of Kentucky, Lexington, KY, United States; ^5^ Radiation Research Program, National Cancer Institute, Bethesda, MD, United States

**Keywords:** radiopharmaceutical, preclinical, validation, nuclear medicine, radiation oncology

## Abstract

Radiopharmaceuticals are reemerging as attractive anticancer agents, but there are no universally adopted guidelines or standardized procedures for evaluating agent validity before early-phase trial implementation. To validate a radiopharmaceutical, it is desirous for the radiopharmaceutical to be specific, selective, and deliverable against tumors of a given, molecularly defined cancer for which it is intended to treat. In this article, we discuss four levels of evidence—target antigen immunohistochemistry, *in vitro* and *in vivo* preclinical experiments, animal biodistribution and dosimetry studies, and first-in-human microdose biodistribution studies—that might be used to justify oncology therapeutic radiopharmaceuticals in a drug-development sequence involving early-phase trials. We discuss common practices for validating radiopharmaceuticals for clinical use, everyday pitfalls, and commonplace operationalizing steps for radiopharmaceutical early-phase trials. We anticipate in the near-term that radiopharmaceutical trials will become a larger proportion of the National Cancer Institute Cancer Therapy Evaluation Program (CTEP) portfolio.

## Introduction

New radiopharmaceuticals intended for clinical use typically follow complex drug-development sequences that expend considerable resources and time. Many are now molecularly targeted, and therefore, might only benefit a subgroup of cancer patients whose tumors express specific targets. Conventional drug-development sequences, which focus on preclinical *in vitro* and *in vivo* studies justifying early-phase I or II trials, and then if warranted, late-phase III trials without assessment of the target expression, are suboptimal in the clinical evaluation of radiopharmaceuticals. Radiopharmaceutical drug-development sequences therefore might benefit from ‘enrichment’ approaches that more reliably reduce patient resources and shorten trial timelines (1). Radiopharmaceutical validation might be considered one of those enrichment approaches.

Validation in oncology means a process whereby preclinical or clinical investigations denote agent performance as being suitable for its intended clinical use (2). For therapeutic radiopharmaceuticals, that agent should demonstrate specificity, selectivity, and deliverability against tumors in a cancer patient subgroup likely to benefit from its prescription. Successful validation improves efficiency in the drug-development sequence by increasing predictive power and by shortening timelines through a fairly small sample size in whom a treatment effect would be expected to be reasonably large (3).

An example of radiopharmaceutical validation is the drug-development sequence for high-specific-activity ^131^I-meta-iodo-benzylguanidine [iobenguane ^131^I (Azedra)] (4–7). Meta-iodo-benzylguanidine (MIBG) is a substrate for a norepinephrine cell surface transporter among chromaffin cells of pheochromocytomas or paragangliomas (4). Unlabeled MIBG disrupts norepinephrine re-uptake, which then disadvantageously lowers tumor uptake of therapeutic ^131^I-MIBG and then harmfully elevates circulating norepinephrine. Elevated circulating norepinephrine induces high-grade acute hypertension (5, 6). Through improved radiochemistry, the iobenguane ^131^I drug product has little to no unlabeled MIBG (7). Phase I and phase II trials were designed to assess up to two dosages of iobenguane ^131^I [500 mCi (or if less than 62.5 kg then 8 mCi kg^-1^)] in the first-line treatment of patients with MIBG-avid pheochromocytoma or paraganglioma, with a primary objective of reduced hypertension (5, 6). In the phase II trial, 25 percent (17 of 68) of patients had reduced use of antihypertension medications (6). This led to regulatory approval of iobenguane ^131^I in MIBG-avid pheochromocytoma or paraganglioma patients 12 years or older (8).

In the National Cancer Institute Cancer Therapy Evaluation Program (CTEP) drug-development sequence, an enrichment approach involves biomarker-driven trial designs for the novel study of radiopharmaceutical agents against cancer. By studying these agents as drugs from the beginning, CTEP staff have adopted the view that radiopharmaceuticals can follow a simpler but leveraged programmatic path for development to meet unmet cancer patient need. For this article, our thoughts are framed by the low- and high-specific-activity tin-117m(4+) diethylenetriaminepentaacetic acid (Sn-117m-DTPA; NSC 824376) radiopharmaceutical, intended first for monotherapy trial evaluation (NCT04616547), and then, combination agent trials. Challenges and opportunities for radiopharmaceutical validation are discussed for Sn-117m-DTPA (created by the nuclear reaction Sn-117(n,γ)Sn-117m; 13.6-day half-life; decays by conversion electrons of 127-129 and 153 keV and by a 158.6 keV gamma photon) (9–15). Here, we propose a four-step process for radiopharmaceutical validation for clinical use. Factors impacting the operationalizing of radiopharmaceutical trials are also discussed.

## Challenges and Opportunities

### Recommended Step 1

For an oncology therapeutic radiopharmaceutical, a sponsor should establish that it is specific, selective, and deliverable against tumors in a patient subgroup likely to benefit from its administration.

Radiopharmaceuticals are drugs that can be infused, ingested, inhaled, or injected. They act as drugs to deliver DNA-damaging radioactivity in the form of alpha-particles (i.e., helium nuclei emitted from the nuclei of radionuclides), beta-particles (i.e., electrons emitted from the nuclei of radionuclides), or conversion electrons (i.e., electrons emitted from electron shells of radionuclides) to cancer cells residing in tumors or circulating in the blood. These drugs are either ‘conjugated’ (i.e., have a ligand-chelator-payload structure) or “neat” (i.e., payload or chelator-payload only). The radiopharmaceutical prostate-specific membrane antigen (PSMA)-targeted thorium-227 conjugate (PSMA-TTC) fits into the conjugate class, as it is given by vein as a slow bolus that then tracks to PSMA-expressing tumors with the aid of the BAY2315493 antibody-chelator ligand (16). In a different way, the radiopharmaceutical Sn-117m-DTPA falls into the neat class. It has two components—a radioactive payload and a radiochemical chelator. Each component adds patient safety risk. Therefore, CTEP has embraced a strategy for drug-development of radiopharmaceuticals that evaluates payload, chelator, and any ligand toxicity prior to embarking on trials. A first step in this strategy is to consider radiopharmaceutical specificity by target antigen immunohistochemistry (IHC) or by microautoradiography.

**Figure 1:**
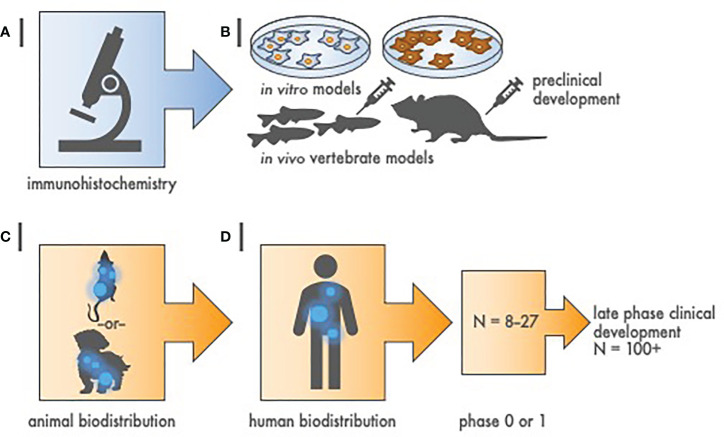
Hierarchy of evidence to justify radiopharmaceutical target-driven early-phase combination trial designs. **(A)** Step 1 of radiopharmaceutical target validation uses tissue microarray (TMA) immunohistochemistry to distinguish tumor antigens of interest from amongst positive and negative controls. **(B)** Step 2 involves two *in vitro* cell survival experiments followed by two *in vivo* tumor growth delay experiments in animal models [e.g., zebrafish (*Danio rerio*) or mouse (*Mus musculus*)]. **(C)** Step 3 is a single rodent or nonrodent species biodistribution study for pharmacokinetics and dosimetry. **(D)** Step 4 is a first-in-human radiation/nuclear medicine imaging experience that precedes early-phase or late phase clinical development. Sample size (N) represents estimated cohort sizes.

Although radiopharmaceutical working groups have enunciated the need, there are no universally adopted guidelines for best practice in determining radiopharmaceutical specificity. The broad variability in investigational zeal and in scientific method for what is used to test radiopharmaceutical specificity is likely responsible for a lack of consensus on a single validation strategy. In our view, the first step should be an IHC assessment of the intended antigen or radiochemical target on formalin-fixed paraffin embedded (FFPE) tissues (**Figure 1**). Our suggested best option for antigen screen is a tissue microarray (TMA). A TMA is a single recipient FFPE block created by extracting multiple cylindrical cores from of interest FFPE donor blocks and re-embedding the cores at defined array coordinates. Several hundred tissue cores can be arrayed into a single FFPE TMA. For a conjugated oncology therapeutic radiopharmaceutical, the nonradioactive ligand-chelator can assay TMA tissue expressing or not expressing the intended cancer cell antigen target. If the ligand-chelator is specific, the IHC stain will be intensely positive on the of interest cancer tissue and null or weakly positive on nontargeted tissue. PSMA-TTC preclinical evaluation is an example where IHC showed target engagement in prostate cancer tumors (16). For a chelator-payload only construct, the IHC method for radiopharmaceutical validation is more challenging. It is most often modelled by postmortem autopsy with scintigrams or microautoradiography. An example using this technique was published for the validation of Sn-117m-DTPA (11). Photography, scintigraphy, and undecalcified microautoradiography showed 47-day posttherapy Sn-117m-DTPA localization of the radiopharmaceutical in mineralizing osteoid interfaces of the thoracolumbar spine from a 64-year-old man with advanced-stage metastatic prostate adenocarcinoma who underwent autopsy 6 h after death (11). Pitfalls to a microautoradiography technique include antigen variability by time to fixation, inadequate fixation period, difference in embalming fixatives, and logistics of calcified/decalcified tissue processing. For these reasons, an appropriately designed TMA, in our opinion, is optimal for antigen screening.

A key to establishing radiopharmaceutical specificity using a TMA is often the correct use of controls. A best-practice negative control is a cell line or tissue that is known not to express the radiopharmaceutical target of interest. Antigen knockout cells, proliferated *in vitro*, pelleted, and then FFPE-cored for the TMA, provide the best negative controls. Antigen nonexpressing cells that are then transfected with the target antigen of interest offer the best positive controls. Given that such cell lines are often beyond the reach of radiobiological laboratories, there are other approaches that can be used to acquire comparable TMA control results. One alternative to antigen knockout cell controls is the use of siRNA or shRNA knockdown cell controls. Another option is to array three or more of interest cancer tissue cores from different patient samples, and then, add at least two corresponding organ normal tissue cores. If the radiopharmaceutical is specific, the IHC stain will be intensely positive on the cancer tissue of interest and null or weakly positive on nontargeted normal tissues in the TMA. Random selection of FFPE donor blocks missing antigen expression, and, antigen heterogeneity are recognized drawbacks of this option, but the cancer core TMA technique was useful in predicting response in uterine cervix cancer (17, 18). A good radiopharmaceutical ligand antigen will have the following characteristics—i) it will bind by IHC to the cancer cell pellets expressing the target antigen, ii) its staining intensity will decrease with increasing dilutions of the IHC ligand, and iii) it will demonstrate an expression pattern among IHC tissues in concordance with biological and mechanistic data.

Our first proposed validation step is relatively quick, inexpensive, and mostly comprehensive. It uses a hierarchy of evidence common in antibody validation (19). While additional research is needed, the demonstration that a radiopharmaceutical is specific, selective, and deliverable against tumors in a patient subgroup likely to benefit from its administration remains essential in the critical evaluation of oncology therapeutic radiopharmaceuticals intended for clinical use.

### Recommendation Step 2

Early-phase oncology therapeutic radiopharmaceutical trials should state a clear, detailed hypothesis, providing pharmacologic or biologic rationale inclusive of at least two cancer models each of *in vitro* and *in vivo* data.

Given that a vast number of radiopharmaceuticals might be evaluated alone or in combination, priority should be given to those that are associated with well-characterized molecular targets, strong mechanistic rationale, and high likelihood for therapeutic success (18). But the level of preclinical data needed to predict therapeutic success in radiopharmaceutical trials is currently unknown (19). Radiobiology *in vitro* and *in vivo* experiments do aid in prioritization of radiopharmaceuticals and their combination partners, and therefore, contribute to the design of biomarker-driven clinical trials (**Figure 1**). We do recognize that there is an evolving use of two-dimensional cell culture (2D) and three-dimensional organoid coculture (3D) model systems, and so, we are reluctant to be authoritative on the modern complexities of radiobiological science. A general preclinical approach might be as follows (20, 21).

First, 2D and now preferably 3D *in vitro* cultures should involve clinically-relevant concentrations and exposures of radiopharmaceuticals alone, or, in combination with oncologic agents. Two cancer cell lines of interest are recommended for *in vitro* experimentation (i.e., two prostate cancer cell lines for a prostate cancer trial proposal) and data should not be extrapolated from unrelated cell lines (20). Cell-derived or patient-derived organoid models might best approximate microenvironmental, oxygenation, or spatiotemporal heterogeneity factors relevant for radiopharmaceutical radiosensitivity (21). We do contend that the actual *in vitro* metabolic or clonogenic survival techniques could vary in this step to assess activity, or here meaning cytotoxic effect size (20). But we advocate that any metabolic cytotoxic “hit” should be confirmed by a clonogenic survival assay, as the latter measures three-to-four logs of cell kill and not just metabolic growth arrest (20, 21). This step might also include assessing activity against radiopharmaceutical and oncologic agent timing (i.e., oncologic agent exposure 1-h before or 1-h after the radiopharmaceutical exposure) or sequence (i.e., radiopharmaceutical first then oncologic agent or the reverse). Here, there is an opportunity for innovation whereby an *in vitro* cell incubator with microfluidic channels for radiopharmaceutical delivery could be manufactured [as was done for low-dose-rate brachytherapy (22)] for experimentation rather than use the usual external beam radiotherapy *in vitro* technique. So overall, we view the first preclinical step as a way to narrow down effective dosages and schedules that will undergo animal model testing. When radioactive dosage and schedule are known in general due to parameters set forth from investigational new drug (IND)-enabling toxicology studies, sponsors might just jump to testing in vertebrate models.

Second, the evaluation of *in vivo* efficacy in vertebrate models for treatment exposures that are relevant in the clinic comes next. Tumor growth delay assays score *in vivo* efficacy by plotting tumor size versus dose to assesses radiopharmaceutical-agent interaction, microenvironment, oxygenation, or spatiotemporal heterogeneity factors (21). We recommend at least two cell-derived or patient-derived *in vivo* models for initial study, best represented by xenografts derived from cancer cells of interest carried forward from *in vitro* experiments (20, 21). A challenge here is whether tumor shrinkage or growth in the vertebrate model system actually predicts human tumor control (21). Take Sn-117m preclinical development (9, 10), where chelators for the Sn-117m were chloride, pyrophosphate, ethyldenehydroxy disodium phosphonate, methylene diphosphonate, or diethylenetriaminepentaacetic acid (DTPA). These chelators display substantial differences for bone uptake, soft tissue uptake, blood clearance, and excretion. As kits for technetium (Tc)-99m diagnostic radiopharmaceutical include milligram quantities of tin(2+) in its stannous form (9), it did not make sense to the sponsor to first expend resources and time in an *in vitro* radiochemical screen. So, in their *in vivo* animal studies, a single strain of Hale-Stoner Brookhaven National Laboratory mice of similar weight and age were injected in the tail vein with two to five microcuries of Sn-117m radioactivity for biodistribution and toxicology studies (9, 10). One mouse experiment involved use of a human osteogenic sarcoma transplant (10). In brief summary, the investigators observed a favorable bone-homing feature of the Sn-117m-DTPA without undue normal organ accumulation or injury from among the radiochemical chelators. Sn-117m-DTPA was selected therefore for clinical development (11–15). We suggest that these Sn-117m-DTPA preclinical studies were mostly comprehensive in that the *in vivo* models i) had all the cellular elements of human tumors (i.e., cancer cells, epithelial and nonepithelial cells, and vasculature) as well as ii) had agent exposure to vital organs whereby toxicity could determine the risk-benefit ratio (9, 10). In general, the second preclinical step justifies dosages and schedules for in human study under an IND.

**Figure 2:**
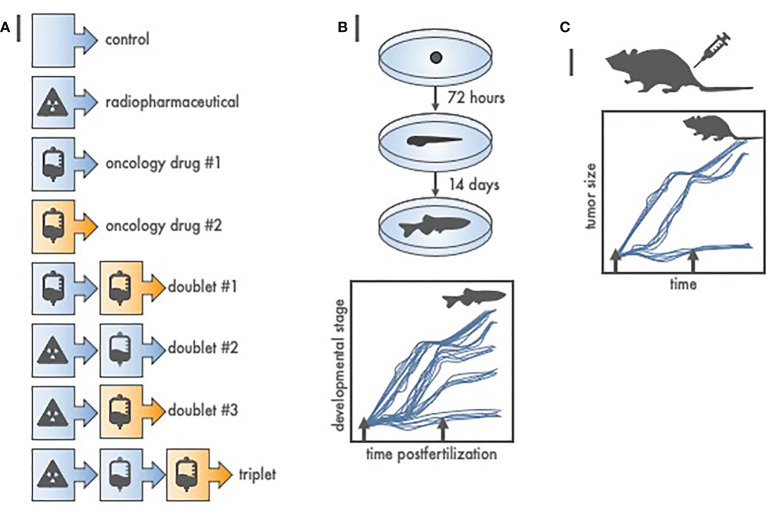
Radiopharmaceutical-drug screen for *in vivo* cytotoxic effect size using zebrafish (*Danio rerio*). **(A)** Listed are at least eight of the possible treatment schemes for a three-agent oncology therapeutic drug screen. To test each treatment in higher order vertebrates (like 40 mice) would be expensive, and, an up to 3-month endeavor. Oppositely, to test each treatment in zebrafish (like 12 fish per treatment from a single 100-embryo clutch) is inexpensive, robust, and finishes in 14 days or less. **(B)** Depicted are basic developmental milestones for zebrafish postfertilization, from embryo to larvae (72 h) to juvenile fish (14 days). Unperturbed zebrafish development is linear. Treatment (arrows) stalls maturation versus time, with greater treatment effect proportional to protracted growth and maturation. Teratogenic treatment effects are also readily apparent. **(C)** Zebrafish-screened “effective” treatments are carried forward to conventional, reduced total animal number, rodent or nonrodent tumor growth delay assays.

Nowadays, we argue that lower vertebrate drug-radiotherapy screens, such as in zebrafish (23), have utility in agent schedule and sequence triaging so that only “effective” treatments are studied in higher vertebrates like rodents, rabbits, or canines. Zebrafish (*Danio rerio*) embryos provide an unusual opportunity to screen drug or radiopharmaceutical agents intended against cancer because of their close genetic and physiologic homology to mammals, their rapid and linear embryonic development in water, their optical clarity allowing inspection of normal organ differentiation and growth, and aqueous environment receptive to radiopharmaceutical, radiotherapy, or oncologic drug exposure. Initial studies associated cytotoxic effect size of radiation response modifiers on defined zebrafish developmental stages postfertilization (23). A zebrafish growth delay assay serves as one example (**Figure 2**). In our example for three oncology drug products, there are at least eight treatment groups. Due to their relatively high clutch size (up to 100 embryos per mating pair) and short linear 14-day embryo-to-larvae-to-juvenile development (24, 25), zebrafish can be used quickly to screen cytotoxic effect size and any unhealthy normal organ toxicity. Despite over three decades of research, an inexpensive zebrafish model remains underutilized in oncology drug product screens prior to rodent or nonrodent pharmacokinetic or toxicology studies.

Regulatory agency guidelines for higher vertebrate safety studies involve comprehensive veterinary observations following radiopharmaceutical dosages in rodents and nonrodents, inclusive of appropriate electrocardiographic measurements in nonrodents (26, 27). The design of toxicology or biodistribution studies in rodents and nonrodents should also assess radioactivity cytotoxicity effect size in vital normal organs. Because of the bone-homing characteristic, hematopoietic deficits resulting from marrow stem cell ablation were an adverse event of special interest in the *in vivo* evaluation of Sn-117m-DTPA (9, 10).

Thus, our step 2 validation process is more laborious, time intensive, and expensive than step 1. But it uses time-honored preclinical *in vitro* and *in vivo* experiments to justify early-phase trials, as has been requested before (20). In our opinion, step 2 offers very strong credentials for evidence indicating benefits of treatment in a radiopharmaceutical-specific subpopulation.

### Recommendation Step 3

An oncology therapeutic radiopharmaceutical used in patients with cancer to treat the disease or palliate tumor-related symptoms should first demonstrate uptake by the tumor and avoid uptake by vital organs in a single animal model species.

Radiation/nuclear medicine follows the lead of the U.S. Food & Drug Administration (FDA) and classifies medical imaging or biological products into at least three categories—contrast agents, diagnostic radiopharmaceuticals, and oncology therapeutic radiopharmaceuticals (26, 27). An image contrast agent is one used to enhance the visualization of tissues, organs, or physiologic processes by increasing the relative intensity of imaging signals in side-by-side body regions. A diagnostic radiopharmaceutical is an agent used in the diagnosis or monitoring of a disease, and, has a radionuclide that decays with an emission of detectable nuclear particles or photons typically linked to a nonradioactive targeting ligand or radiochemical carrier. Such agents are used in nuclear medicine procedures like planar imaging single photon emission computed tomography (SPECT), positron emission tomography (PET), or in combination with other emitted radiation detectors. A therapeutic radiopharmaceutical is usually a ligand- or radiochemical-chelated radionuclide used in the treatment of a disease or in the palliation of disease-related symptoms (e.g., pain). Such agents are used in radiation medicine/oncology treatments. The term “theranostic radiopharmaceutical” means the same nonradioactive targeting ligand or radiochemical chelator carries either i) a diagnostic radionuclide to assess tumor uptake and any spread of cancer elsewhere in the body, or ii) a therapeutic radionuclide as treatment (28). We forecast that next generation radiopharmaceuticals likely will bear both a diagnostic and a therapeutic radionuclide at the same time. Step 3 in our process first involves showing oncology therapeutic radiopharmaceutical uptake by the tumor and avoidance of vital organs in a single animal species (**Figure 1**).

Standardized radiation/nuclear medicine practice evaluates post-administration radioactivity in organs over time intervals (like 5 × the effective half-life). This creates time-integrated activity curves. Organs measured for time-integrated activity include the adrenal glands, bone and bone marrow, brain, small and large intestine walls, stomach, heart, kidneys, liver, lungs, muscles, ovaries, pancreas, spleen, testes, thymus, thyroid, urinary bladder, uterus, and total body (26, 27). Tabular data for absorbed dose estimates should be reported, as has been done for Sn-117m-DTPA (12). Radioactivity in urine and in feces should be recorded for elimination pharmacokinetic assessments. If adequately justified, the number of organs evaluated might be abbreviated to include bone marrow and organs of excretion, such as kidneys and liver, due to these organs being exposed regardless of target binding. An example of using a limited biodistribution organ list is when demonstrating comparability of two related oncology therapeutic radiopharmaceuticals (bridging study) such as a switch from a low-specific activity to high-specific-activity product like is planned for Sn-117m-DTPA. In our view, the experimental design of an animal biodistribution study should integrate, when possible, medical components of any planned early-phase human biodistribution and dosimetry study, as these medical components might affect the eventual distribution of a radiopharmaceutical product. For example, if a planned early-phase clinical trial allows patients being pretreated by bone health agents (e.g., zoledronic acid or denosumab) to enroll, then the use of bone health agents should be considered in the animal biodistribution study. This consideration is often omitted in favor of simple and efficient single dose animal imaging study in a single species (26, 27).

**Figure 3:**
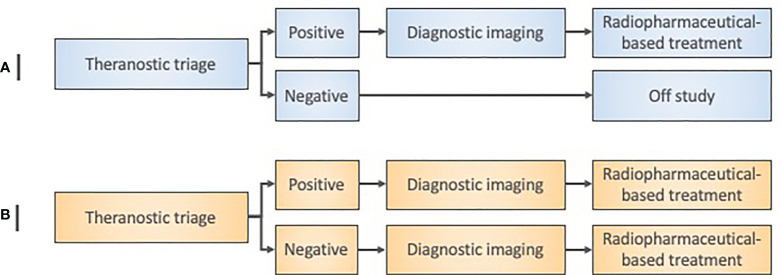
Radiopharmaceutical target-driven early-phase enrichment trial designs. **(A)** Early-phase 0 enrichment trial designs evaluate a new treatment only in a theranostic target-positive subject subpopulation. **(B)** Early-phase I trial designs for agent safety as a primary objective in an ‘all-comer’ approach might otherwise assign both target-positive and target-negative patients to a radiopharmaceutical treatment under investigation. Diagnostic imaging means baseline and posttherapy conventional radiation/nuclear medicine imaging for initial exploratory objective response assessment in either trial design.

**Figure 4:**
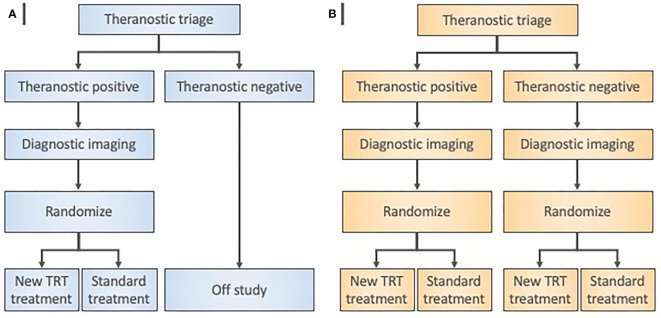
Radiopharmaceutical target-driven phase II combination trial designs isolating treatment effects. **(A)** Enrichment phase II trial designs evaluate a diagnostic agent in a theranostic pair as a triage step in all patients. Random allocation applies only to patients with “theranostic-positive” results. **(B)** Theranostic pair-stratified designs randomly allocate both “theranostic-positive” and “theranostic-negative” patients to the radiopharmaceutical-based treatment under investigation. Diagnostic imaging means baseline and posttherapy conventional radiation/nuclear medicine imaging for objective response assessments.

In our view, step 3 determines whether there are any body sites in which the radioactive drug particularly concentrates, or, in which the radioactive drug predominantly excludes. In our opinion, step 3 ties together the preclinical data from step 1 and step 2 into a programmatic platform sufficient to test a new oncologic therapeutic radiopharmaceutical in a subgroup of “theranostic-positive” patients (**Figures 3**, **4**). This step will provide an estimate of radiation doses that could be delivered to the tumor without exceeding the maximum safe doses to normal tissues.

### Recommendation Step 4

An oncology therapeutic radiopharmaceutical used in patients with cancer to treat the disease or palliate tumor-related symptoms should demonstrate uptake by the tumor and avoid uptake by vital organs in a first-in-human microdose study.

Our fourth recommended validation step provides visual and physical evidence, in a conventional first-in-human microdose study, that a possible oncology therapeutic radiopharmaceutical demonstrates uptake by the tumor and avoids uptake by vital organs. Currently, the microdose study can be conducted under an Exploratory Investigational New Drug (xIND) application, as outlined in a 2006 FDA guidance (29). In these first-in-human studies, very small single doses (e.g., 1/100^th^) of an agent are administered for medical imaging assessment, and therefore, the hazard for toxicity is rare. We argue that a conventional microdose study informs the evaluation of oncology therapeutic radiopharmaceuticals by providing more data on human i) biological effects, ii) starting doses, and iii) schedules. Microdose studies are an important precursor to phase 0 or phase I trial design in that they might inform patient selection or biospecimen sampling strategies (**Table 1**).

**Table 1:** Microdose study elements that inform radiopharmaceutical phase 1 or phase 0 trials.

Microdose Study Element	Phase 1 trial design impact	Phase 0 trial design impact
Primary biodistribution endpoint	Sets dosage recommended for phase 1 study	Sets dosage providing target modulation
Biomarker assay	Adds exploratory pharmacodynamic assay	Adds integral pharmacodynamic endpoint
Bioimaging or dosimetry	Adds exploratory pharmacodynamic assay	Adds integrated pharmacodynamic endpoint
Dose dilution sub-study	Identifies CTCAE toxicity of special interest	Sets dosage for desired target modulation
Dose administration	Rationalizes multiple cycle administration	Justifies single cycle administration
Single species safety evaluation	Rationalizes 15–18 patient study	Justifies 8–10 subject study
Pharmacokinetics	Provides batched blood sampling frequency	Provides real-time blood sampling frequency

CTCAE, Common Terminology Criteria for Adverse Events (current version).

Step 4 in our view uses the same ratio of nonradioactive-to-radioactive components that was used in the step 3 single animal species study. Following our example, the low-specific-activity Sn-117m-DTPA has a 20-fold molar excess of the acid salt of DTPA over radioactive Sn-117m (12). The same 20:1 low-specific-activity product that was given in rodent and nonrodent SPECT biodistribution studies was given in a first-in-human anterior-posterior single-pass 18-min whole-body SPECT scan (10 cm/min with matrix 1024 x 512). Sn-117m-DTPA human bone activity was about constant over a 196-h observation period; Sn-117m-DTPA blood activity nearly completely disappeared in the first 15 min post-administration (12). Sn-117m-DTPA SPECT images were comparable to Tc-99m-methyl diphosphonate (MDP) scintigrams (12). Such pharmacokinetic and pharmacotoxic data inform next step trials (**Figures 3**, **4**). Microdose study data inform “theranostic triage” for trial enrichment, whereby a positive imaging study (e.g., SPECT) assigns trial-specific treatment rather than to all. Phase II trials utilize enrichment by selecting only theranostic triage-positive patients, and then, randomizing trial-specific or standard treatment.

Step 4 provides clinical experience upon which other early-phase trials of an oncology therapeutic radiopharmaceutical test safety and efficacy. It will also verify the estimate of radiation doses that could be delivered to the tumor without exceeding the maximum safe doses to normal tissues from step 3. In many clinical scenarios, step 4 microdose studies have been done at the outset of the drug-development sequence. However, we strongly encourage progressing through steps 1 to 4 in sequence as the overall process provides the best justifications for human trials as well as provides a relative cost-effective and timely hierarchy of evidence.

## Perspectives on Operationalizing Radiopharmaceutical Early-Phase Trials

Certain new therapeutic radiopharmaceuticals like high-specific activity Sn-117m-DTPA use radionuclides in drug products that are not yet approved for clinical use by the FDA. In this case, there has been a discussion of the usefulness of modular drug master files (DMF) that draw upon components of other FDA-filed DMFs for radiochemistry, manufacturing, and control processes (30). This ensures patient safety and speeds up IND-enabling studies that justify early-phase trials.

In the current early-phase operationalization process for radiopharmaceutical IND-sponsored trials, trial sites must submit their radioactive materials license (RML) on a per study basis to indicate its authority to handle a specific radionuclide drug product for medical use, as guided by the Nuclear Regulatory Commission (NRC) and applicable agreement state regulations. The site’s RML (updated as needed for new radionuclide authorizations, authorized users, or license expirations) is used by CTEP to credential the trial site as a targeted radiopharmaceutical facility organization (TRF). Sites desiring to enroll patients to CTEP-sponsored radiopharmaceutical trials must align themselves with their TRF *via* the Clinical Trials Support Unit (CTSU) regulatory provider association function. An attestation that the site uses a valid calibrated dose calibrator for measuring the relevant isotope radioactivity is a necessity. A list of all authorized users of a specific radionuclide under clinical investigation must be provided to CTEP and verified against the site RML for authorized user credentialing (inclusive of nuclear medicine and radiation oncology physicians, radiation or nuclear medicine physicists, technicians, or radiopharmacists involved in prescribing, receipt, storage, handling, preparation, dispensing, and treatment of study patients with radiopharmaceuticals). Depending on the radiopharmaceutical under investigation, radionuclide-specific training on safe handling and clinical use must be documented either by the RML or radionuclide training certificate verified by CTEP. To illustrate these points, take the training requirement for handling Sn-117m-DTPA. Training is not required by U.S. Federal law or FDA regulation, but the knowledge and experience gained by site authorized users on safe handling and clinical use is a state regulation. It is also good clinical practice. We anticipate such training to be documented at each trial site before any accrual to an early-phase trial of Sn-117m-DTPA.

Another key aspect for radiopharmaceutical CTEP IND-sponsored trials tracks investigator and sub-investigator trial tasks on the clinical trial delegation of tasks log (DTL). New task assignments for radiopharmaceuticals are required on a CTEP IND-sponsored trial DTL. First, the DTL must list two or more authorized user physician prescribers for the radiopharmaceutical as identified by the clinical trial site’s RML or Radiation Safety Officer (RSO) approved list. Second, the DTL must identify at least two trained and authorized persons for the task of receipt, storage, handling, preparation, dispensing and final disposition of the radiopharmaceutical (again as identified by the clinical trial site’s RML or RSO approved list). Any other sub-investigators involved in the clinical investigation (both authorized users for medical use and other site trained personnel) must also be identified on the site DTL. CTEP intends to work on ways to facilitate the site and investigator/sub-investigator credentialing process for radiopharmaceutical trials, to eventually reduce any duplicity in the credentialing process across clinical investigations. Further effort in enhancing collection of site credentials and authorized personnel site rosters is needed.

## Conclusion

In summary, this article explores elements in radiopharmaceutical validation for clinical use as they relate to the justification of early-phase clinical trials. It sharpens thinking about the levels of evidence used to predict radiopharmaceutical therapeutic success through a discussion of trial-enabling immunohistochemistry, preclinical *in vitro* or *in vivo* experiments, biodistribution and dosimetry studies, as well as first-in-human experience. Education on the operationalizing of radiopharmaceutical trials for authorized users and their support staff remains essential for the favorable development of these forms of cancer treatment. Delegation of task logs are key elements in the responsible conduct of early-phase radiopharmaceutical trials for therapeutic intent.

## Author Contributions

All authors contributed to the article and approved the submitted version.

## Conflict of Interest

The authors declare that the research was conducted in the absence of any commercial or financial relationships that could be construed as a potential conflict of interest.
